# Identification of TIM3 2′-fluoro oligonucleotide aptamer by HT-SELEX for cancer immunotherapy

**DOI:** 10.18632/oncotarget.6608

**Published:** 2015-12-14

**Authors:** Sandra Hervas-Stubbs, Mario M. Soldevilla, Helena Villanueva, Uxua Mancheño, Maurizio Bendandi, Fernando Pastor

**Affiliations:** ^1^ Program Immunology and Immunotherapy, Centro de Investigaciones Medicas Aplicadas (CIMA), Pamplona, Spain; ^2^ Instituto de Investigación Sanitaria de Navarra (IDISNA), Recinto de Complejo Hospitalario de Navarra, Pamplona, Spain; ^3^ Program of Molecular Therapies, Aptamer Unit, Centro de Investigaciones Medicas Aplicadas (CIMA), Pamplona, Spain; ^4^ Ross University School of Medicine, Portsmouth, Commonwealth of Dominica

**Keywords:** immunotherapy, cancer, exhaustion, aptamer, therapeutics

## Abstract

TIM3 belongs to a family of receptors that are involved in T-cell exhaustion and Treg functions. The development of new therapeutic agents to block this type of receptors is opening a new avenue in cancer immunotherapy. There are currently several clinical trials ongoing to combine different immune-checkpoint blockades to improve the outcome of cancer patients. Among these combinations we should underline PD1:PDL1 axis and TIM3 blockade, which have shown very promising results in preclinical settings. Most of these types of therapeutic agents are protein cell-derived products, which, although broadly used in clinical settings, are still subject to important limitations. In this work we identify by HT-SELEX TIM3 non-antigenic oligonucleotide aptamers (TIM3Apt) that bind with high affinity and specificity to the extracellular motives of TIM3 on the cell surface. The TIM3Apt1 in its monomeric form displays a potent antagonist capacity on TIM3-expressing lymphocytes, determining the increase of IFN-γ secretion. In colon carcinoma tumor-bearing mice, the combinatorial treatment of TIM3Apt1 and PDL1-antibody blockade is synergistic with a remarkable antitumor effect. Immunotherapeutic aptamers could represent an attractive alternative to monoclonal antibodies, as they exhibit important advantages; namely, lower antigenicity, being chemically synthesized agents with a lower price of manufacture, providing higher malleability, and antidote availability.

## INTRODUCTION

The recent approval of immune-checkpoint blockade strategies has underscored the importance of the immune system in the control of tumor growth [1–3]. In most cases, the tumor induces a persistent chronic immune response that triggers the exhaustion of tumor-reactive lymphocytes that turn incapable of responding to the tumor antigens [4]. A feasible strategy is to block the receptors that lie underneath T-cell exhaustion, which has been shown to restore T-cell function [5]. Among the exhaustion associated to T-cell receptors, we should highlight three: PD1, TIM3 and LAG3 [6, 7]. The result in clinical trial with the blockade of PD1 has been unprecedented in aggressive antigenic tumors such as melanoma. In pre-clinical studies the blockade of the PD1:PDL1 axis together with any of the other two receptors (TIM3 or LAG3) has been broadly documented to exhibit synergistic antitumor effects [8, 9].

TIM3 expression was initially identified in CD4 IFN-γ producing cells and in cytotoxic CD8 lymphocytes [10]. The blockade of the TIM3 has been shown to exacerbate autoimmune diseases associated with Th1 responses, underscoring the role of the receptor in holding back the immune response [10]. TIM3 and PD1 are co-expressed by the most dysfunctional and exhausted T cells [10]. Contrary to PD1, the inhibiting signal of TIM3 is not mediated by ITIM cytoplasmic tails [11], which indicates that the blockade of both immune-checkpoints will not display overlapping effects. Interestingly, several preclinical studies have shown that combined targeting of the TIM3 and PD1 pathways is more effective in controlling tumor growth than targeting either pathway alone [8]. TIM3 is also expressed in a subpopulation of Treg that has been specially enriched in tumor infiltrates – they show a more potent immunosuppressive capacity and their presence correlates with a worse prognosis in cancer patients [12]. TIM3 also has a role in tumor-associated macrophages (TAM) and tumor Dendritic Cells, being up-regulated in these types of cells [12]. The ligand of TIM3 has been initially identified as galectin-9 [13]. However, there are some recent publications indicating that there might be other ligands that still need to be identified [14].

There are currently several clinical trials with immunotherapeutic approaches that are aimed at reverting T-cell exhaustion, most of them focused on intervening in the PD1/PDL1 axis. Nevertheless, in spite of all the promising recent data in preclinical models, TIM3 has not yet been evaluated in clinical trials.

Aptamers are single-stranded oligonucleotide molecules that are selected through a complex combinatorial process named Systematic Evolution of Ligands by Exponential Enrichment (SELEX) [15]. Selected aptamers can be chemically synthetized, facilitating the GMP production. They display other important advantages when compared with monoclonal antibodies, e.g. great malleability, low antigenicity, high penetration rate (as they are small molecules), and, in case of side effects, the possibility of neutralizing every aptamer activity *in vivo* with an antidote [16].

## RESULTS

### Identification of TIM3 aptamer by HT-SELEX

**Figure 1:**
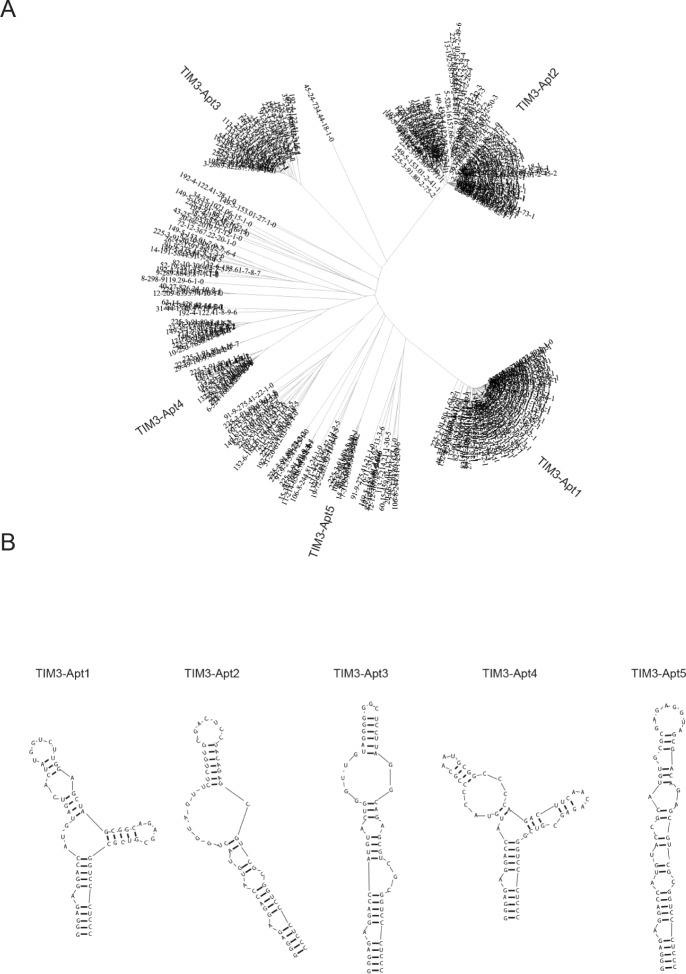
Major TIM3 aptamer families identified by HT-SELEX **A.** The sequences of aptamer identified from round 6 were HT-sequenced by Ion Torrent, the sequencing alignment was performed with FASTAptamer software and then they were clustered by using the ClustalW. **B.** Secondary structure predicted by using RNAstructure of the five most abundant aptamer families.

TIM3 aptamers against the chimera murine recombinant protein TIM3-Fc was performed by SELEX and high-throughput sequencing. We initiated the selection with a 25N-nucleotide library, shorter than usual, to avoid further truncation steps after the aptamer identification. The random regions were flanked by two constant sequences that were added in order to transcribe the DNA library into RNA and to amplify the selected species by PCR in each round. The selection was performed with 2′ fluoro-pyrimidine bases in order to increase the RNA stability and the resistance to RNAse degradation. The screening selection was done against murine TIM3-Fc recombinant protein chimera. Counter-selection against IgG1 was performed before each round of SELEX to remove all the aptamers that might bind to the Fc domain. The aptamer binding was performed at physiological buffer and at 37°C, with increasingly restrictive conditions in each round. The aptamer selection was stopped at round 6 to identify the enriched species by last generation of sequencing (Ion Torrent). The analysis was performed by using the FastAptamer software (Figure 1). FASTAptamer analysis was able to identify other minor families of aptamers (Supplementary Data 1). The aptamers that were recognized by FASTAptamer were clustered with ClustalW software (Figure 1A), identifying more than 5 major families of TIM3 aptamers (Figure 1B). Out of all the families we chose the two that were most highly amplified in the selection process, TIM3-Apt1 and TIM3-Apt2, which were enriched at 231.072 and 153.681 reads per million respectively (Supplementary Data 2).

### TIM3-Apt1 and TIM3-Apt2 bind to rmTIM-3-Fc protein with high affinity

**Figure 2:**
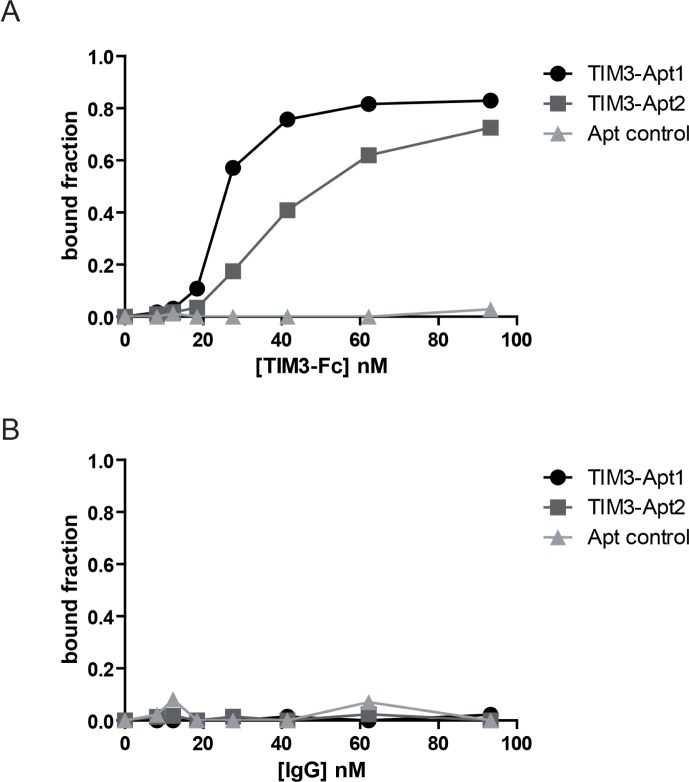
Binding of the two most abundant TIM3 aptamers to the mouse recombinant protein TIM3 **A.** Binding of TIM3-Apt1 TIM3Apt2 performed by filter-binding assay described in methods to the chimeric recombinant protein mTIM3-Fc; a randomized Apt control library was used as a negative binding control. **B.** Binding of TIM3-Apt1, TIM3-Apt2 and Apt-control to IgG1 protein.

The most abundant aptamers during the selection, TIM3-Apt1 and TIM3-Apt2, were chosen for further characterization. The secondary prediction of the aptamer is shown in Figure 1, generated by the software RNAstructure 5.3. We selected the sequence structures with lower energy. They do not share any preserved motives, which indicates that they might be binding to different aptatopes. The affinities of each aptamer to TIM3-Fc recombinant protein were performed by filter-binding assay as previously described, and the apparent Kd of each aptamer was 22 nM for the TIM3-Apt1 and 40 nM for the TIM3-Apt2 [17]. An irrelevant aptamer was used as control. No binding to IgG1 was observed that could foreclose the possibility that the aptamers might be binding to the TIM3 extracellular motive instead of binding to the Fc (Figure 2). Despite 60% homology of murine TIM3 and human TIM3, the TIM3-Apt1, which showed a higher inhibition rate, did not bind to the human TIM3 protein, which suggests the high specificity of this aptamer (data not shown). Lack of binding to the human recombinant protein TIM3-Fc, which displays the same IgG1 Fc domain and linker, indicates that the aptamer TIM3-Apt1 is indeed binding only to the mouse TIM3 domain.

### TIM3 RNA aptamers recognize mouse TIM3 on the cell surface

**Figure 3:**
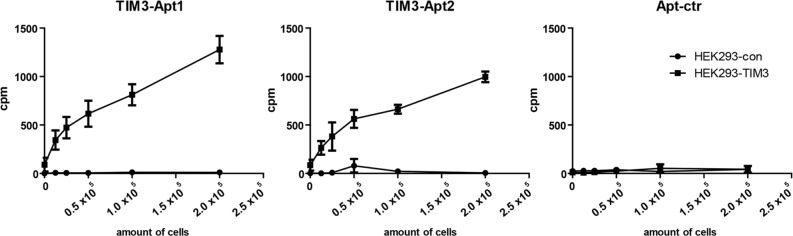
Binding of TIM3 aptamers to surface-expressing TIM3 cells TIM3-Apt1, TIM3-Apt2, and Apt-ctr labeled with P32 as described in methods to different amount of cells transfected with TIM3 cDNA or to the parental cell line.

Based on the affinity and the abundance of the aptamers selected, we focus on the further characterization of TIM3-Apt1 and TIM3-Apt2. The fact that the aptamers bind to the recombinant protein does not necessarily mean that the aptamers would bind to the native TIM3 protein expressed on the cell surface. In order to validate the binding of the TIM3-Apt to the extracellular motive of TIM3 expressed on the cell surface, we generate a TIM3 cDNA plasmid stably transfected cell line, HEK-293-TIM3ext, which expresses the mouse TIM3 extracellular motive (Supplementary Figure 1). To avoid any structural modification on the aptamer associated with fluorochrome labeling, which could derive in loss of aptamer binding, we spiked the aptamer transcription with αP32-ATP. The P32 radioactive TIM3-Apts were used to determine the binding to HEK-293-TIM3ext cells; HEK-293 parental cells were used as a negative control of the binding assay, as well as an aptamer control (Apt-ctr). To prove that the binding was dependent on the TIM3 expressed on the cells, we performed serial dilutions of the cells. As we can see in Figure 3, the TIM3-Apts bind only to the HEK-293-TIM3ext cells and not to the parental ones. The binding is diminished proportionally with each dilution; the detection limited by centillation counter is reached at 12.5×10^3^ cells.

### TIM3-Apt1 counteracts TIM3 inhibitor signal on T lymphocytes

**Figure 4:**
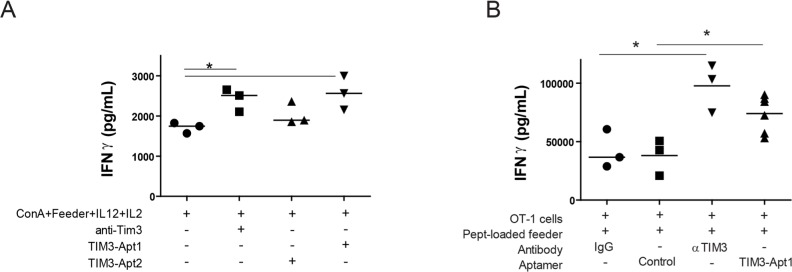
TIM3-Apt1 antagonizing aptamer boosts IFN-γ production in CD8 lymphocytes **A.** Purified CD8 lymphocytes were polyclonally stimulated with ConA, feeder splenocytes and IL12 and IL2 cytokines. Those lymphocytes were at the same time treated with anti-TIM3 monoclonal antibody (RMT 3-23), or TIM3-Apt1 or TIM3-Apt2, and INF-γ production was quantified by ELISA in the supernatant after 72 h of incubation. **B.** Purified CD8 lymphocytes from OT-I lymphocytes were activated with preloaded SIINFEKL-peptide splenocytes, IL12 and IL2 cytokines together with the anti-TIM3 monoclonal antibody, or the TIM3-Apt1 antagonist, or an Apt-control.

In order for an aptamer to display a biological effect, it is not only necessary for the aptamer to bind to the receptor but it needs to disrupt the transduction signaling of the receptor. Therefore, in order to determine whether the aptamer would be able to revert T-cell exhaustion *in vitro*, we isolated CD8 lymphocytes from the spleen of WT mice and these cells were stimulated with Concanavalin A, IL-12 and IL-2 in the presence of feeder splenocytes for four days. Under these conditions CD8 T lymphocytes upregulated the expression of TIM3 and PD1 (Supplementary Figure 1), indicating that they are acquiring inhibitor cell markers. The addition of TIM3 blocking reagents, such as the anti-TIM3 Ab (RMT 3-23) or the TIM3-Apt1 significantly increases the secretion of IFN-γ measured by ELISA (Figure 4A). TIM3-Apt2 does not show any immune-stimulatory effect, which could be due to the lower affinity of this aptamer to the TIM3 receptor or just because it binds to a different aptatope that does not preclude the binding of TIM3 to its ligand. Another experiment was performed with CD8 lymphocytes from OT-I transgenic mice (whose TCR recognized the determinant antigen SIINFEKL derived from ovalbumin) were co-cultured with SIINFEKL-peptide loaded feeder splenocytes (Figure 4B). The level of IFN-γ produced by OT-I lymphocytes stimulated in the presence of TIM3-Apt1 was slightly lower than that of OT-I cells stimulated in the presence of the bivalent anti-TIM3 mAb, although both groups reached comparable statistical significance. Neither the isotype IgG control nor a scramble control aptamer triggered the production of IFN-γ (Figure 4B). These results indicate that the TIM3-Apt1 is able to antagonize the TIM3 receptor on T lymphocytes, thus enhancing their effector function as measured by IFN-γ production.

### TIM3-Apt1 reduces CT26 tumor burden in combination with PDL1 blockade

**Figure 5:**
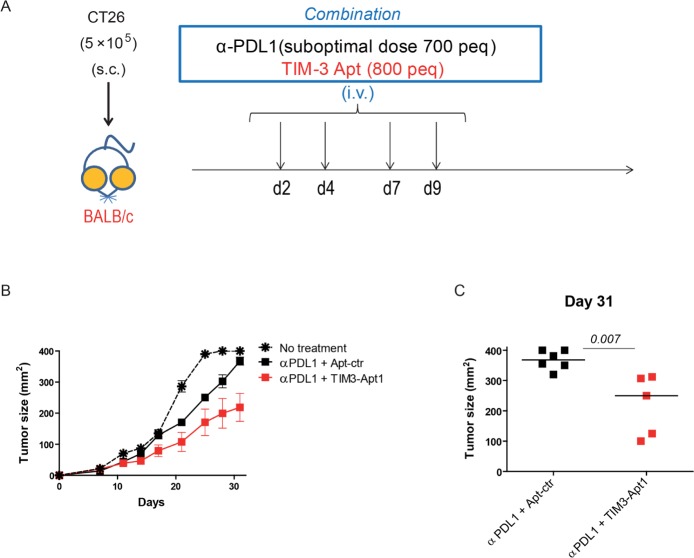
TIM3-Apt1 antagonizing aptamer enhances tumor-bearing mice in combination with PDL1 blockade **A.** CT26 tumor colon carcinoma was implanted subcutaneously in Balb/c mice; at day 2, 4, 7, 9 upon tumor implantation mice were treated intravenously with PDL1-blocking antibody (10F.9G2) and the TIM3-Apt1 antagonizing aptamer and tumor volume was monitored by caliber measurement. **B.** Tumor growth kinetics of mice treated with anti-PDL1 antibody and TIM3-Apt1 or anti-PDL1 antibody with Apt-control. **C.** Tumor size at day 31 upon tumor implantation of the mice treated with PDL1 antibody and TIM-Apt1 blockade or just with anti-PDL1 antibody and Apt-control (*p*=0.007) (*n*=5-6 mice per group).

The disruption of the TIM3-signaling axis in combination with PD1:PD1L blockade has been shown to induce a remarkable antitumor effect in several tumor models in pre-clinical settings [8]. In order to prove that the selected anti-TIM3 aptamer is able to trigger immune mediated antitumor responses in combination with PDL1 blockade, we used the colon carcinoma orthotic tumor model CT26 implanted subcutaneously in Balb/c mice. CT26 tumor model has been described to trigger T-cell exhaustion with upregulation of TIM3 [8]. We treated the mice after tumor inoculation at day 2, 4, 7, 9 with 800 pmols/per injection intravenously of TIM3-Apt1 or aptamer control (Apt-con) together with 350 pmols (700 peq) of anti-PDL1 antibody (10F.9G2) as indicated in Figure 5A. The combination of TIM3-Apt1 and PDL1-blocking antibody reduced the tumor burden significantly in comparison with the PDL1 blocking monotherapy, which did not display such a remarkable antitumor effect. The TIM3-Apt1 as monotherapy treatment at day 2, 4, 7, 9, 11 and 13 with 800 pmol per injection did not display an antitumor effect (Supplementary Figure 3). It is noteworthy that no signs of discomfort that could be associated with toxicity were observed in any of the treated mice, suggesting that the treatment was well tolerated.

## DISCUSSION

The immune response is tightly regulated at cellular level by several mechanisms triggering either activation or inhibition signals. The balance between those signals determines the induction of specific immunity avoiding collateral damage which could derive in autoimmunity. In chronic inflammation, as it occurs with some pathogens and cancer, the effector function of the reactive lymphocyte is hampered, turning those lymphocytes into an exhausted phenotype [4]. The molecular mechanisms that underlie this cell transformation are complex, but we can highlight three important cellular receptors that play a key role in this process: PD1, TIM3, and LAG3. The development of agents to block those receptors allows for therapeutic intervention to counteract T-cell exhaustion. Most therapeutic agents to counteract T-cell exhaustion, currently in clinical trials or already approved by the FDA (nivolumab for PD1), are monoclonal antibodies or cell-derived protein products. The uses of these agents that are broadly used in clinical settings still have important limitations: I) The repeated administration of protein therapeutic agents, even with fully humanized monoclonal antibodies, can trigger a neutralizing T-cell dependent humoral immune response, significantly reducing the effect of the treatment or causing other side effects; that could be especially important in cancer relapsing patients who would need to re-initiate the same protein-agent treatment [18–20]. II) Cell-derived products imply a high manufacture cost and a complex regulatory process for clinical applications, showing staggering market prices which in the end reduce the availability of this type of treatment for most cancer patients. Furthermore, it has been shown that the combination of different immune-checkpoint blockades significantly enhances therapeutic efficacy, indicating that in the future it would be desirable to combine several immunotherapeutic agents, which would in turn raise even more the market prices for this type of treatments. III) Side effects associated with exacerbated immune responses triggered by immune-checkpoint blockade could derive in severe auto-reactive immune responses. Monoclonal antibodies have long half-lives and no antidote is available. Therefore, in case of side effects, the only way to proceed in order to ameliorate the self-reactive immune response is to initiate a treatment with potent immunosuppressive drugs, while the monoclonal antibody is still present in the blood stream without been neutralized [18]. Presumably, though the combination of several therapeutic agents would improve the antitumor immune response, it would also trigger more serious auto-inflammatory immune responses in the patients. It is urgent to develop a more reliable contingent plan to counteract side effects associated with this type of treatments that will not elicit potent immunosuppression in cancer patients.

Aptamers are emerging in the therapeutic arena as an alternative to antibodies with significant translational advantages. They display similar to or even superior affinity and specificities than antibodies, but they are chemically synthesized drugs, reducing the manufacturing costs for clinical application [21, 22]. Besides, aptamers are very malleable molecules than can be easily modified [21] to change their pharmacokinetics according to the needs of the treatment, either to enhance or reduce the half-life of the therapeutic agent in the blood. There is always an antidote available for each aptamer in case of side-effect signs, which will neutralize aptamer activity *in vivo* within minutes upon systemic administration [16, 23, 24]. In the last few years several immune-modulatory aptamers have been developed to enhance or block immunity [25]. They have been used in cancer immunotherapy settings, acting as agonists of costimulatory molecules (4-1BB, OX40, CD28, CD40) [26–29] blocking inhibitor signals (CTLA4, PD1) [30, 31], or reducing immunity in transplant and autoimmunity models (CD200) [30].

In this work we identified the first-in-class TIM3 antagonistic aptamer by HT-SELEX. Deep sequencing allows for the identification of over five different aptamer families. We performed the binding of only the two most frequent families, and tested the biological and therapeutic effects of the most abundant one. The most abundant aptamers might not always be the ones with higher affinity; however, considering the amount of families that we identified, we decided to focus on the two most populated aptamer families. In further experiments, we will try continue the SELEX from round 6 by altering the selection against TIM3-Fc human recombinant protein to try to identify cross-reactive aptamers as recently described [32]. The two aptamers we chose for further characterization showed high affinity and specificity to their targets. TIM3 blockade, as previously described, is able to potentiate the production of IFN-γ cytokine as an indicator of T-cell exhaustion reversion. TIM3-Apt1 was able to enhance the IFN-γ production of CD8 polyclonal activated T cells and on TCR-specific CD8 OT-I activated lymphocytes similar to the bivalent TIM3 antagonist antibody. On a therapeutic setting, the antagonistic aptamers synergized with suboptimal doses of PDL1 blocking antibody, displaying an antitumor therapeutic effect at very low doses of both antagonistic molecules (PDL1 and TIM3). This is the first generation of TIM3 aptamers and it has shown a remarkable antitumor activity in its monomeric form *in vivo*. Multimerization and/or pegylation as previously done [30, 31] would presumably improve the antitumor effect significantly, and might even surpass the effect of the antibody.

## MATERIALS AND METHODS

### Aptamer selection

The SELEX procedure, as previously described [27], was used for the selection of aptamers against a chimera-recombinant protein that contains the extracellular mouse TIM3 domain fused to human IgG1-Fc (R&D systems, Abingdom, UK). A 25N-nucleotide randomized DNA library flanked with two constant regions was used as a library template GGGAGGGACCGCGAC GCTCTGNNNNNNNNNNNNNNNNNNNNNNNNNTA CATGGTCCTCTCCC; the primers to amplify the library were Fwd GGGGAATTCTAATACGACTCACT ATAGGGAGAGGACCATGTA and Rev GGGAGGGA CCGCGACGCTCTG. In order to increase RNA stability and to confer resistance to RNAses, we used 2-Fluoro-UTP and 2-Fluoro-CTP modification. *In vitro* transcription was carried out with Durascribe kit (Epicentre, Madison, WI, USA). The selection conditions that were used in each round of SELEX are described in Supplementary Table 1. The selections were carried out at 37°C in saline buffer (20mM HEPES, 150 mM NaCL, 2mM CaCl2 and 0.01% BSA) in rotation for 30 minutes. mTIM3-Fc chimera recombinant protein was bound to protein A sepharose (GE Healthcare Bio-science, Upssala, Sweden) beads, and precipitation was used to isolate the RNA bound fraction. The RNA bound fraction was recovered via phenol-chloroform-isoamyl alcohol extraction, followed by reverse transcription, PCR and transcription, as previously described [27]. We performed counter-selection against human IgG1-bound A sepharose beads before each round of SELEX (Sigma Aldrich, Saint Louis, MO, USA). In order to remove any RNA bound during the counter-selection that may have become detached from the pre-clearing sepharose protein A beads, the supernatant from the pre-clearing step was incubated with a nitrocellulose disk (Whatman, Dassel, Germany) at room temperature for 10 minutes.

The DNA-PCR products of round 6 were HT-sequenced by Ion Torrent, and multiple-sequence alignment was performed by using FASTAptamer software [33] to get the FASTAcount file which indicates the number of reads per million of each selected aptamer and the ranking. With this file we performed a FASTAcluster which was set at a -d 7 -f 2 to select only the aptamer families that were more abundant. The result of FASTAcluster was again clustered with ClustalW and visualized with Seaview software. All the process was done in a Linux CentOS 6.3 cluster of 4 cores and 64GB. RNA-structure predictions were performed with RNAstructure 5.3 software.

As Apt-ctr in all the experiments we have used a randomized 2′F-RNA 25N aptamer flanked with the same constant regions at 5′ and 3′ than the TIM3 aptamers.

RNAs aptamer affinity for the recombinant TIM3–Fc was measured by a double-filter nitrocellulose filter-binding assay [17] with some modifications [34].

### Cell binding

The TIM3-Apt1 and TIM3-Apt2 were radio labeled by spiking P32-αATP in each transcription reaction. To assess the binding of the radioactive aptamer, we generated a stably transfected cell line which expressed the extracellular domain of TIM3 by using the Addgene plasmid 49208[35]. The stably transfected 293 cell line was incubated with the labeled radioactive aptamers, at different cell ratios; as negative control we used the non-transfected parental cell line.

### Mice

C57BL/6 and BALB/c mice were from Harlan (Barcelona, Spain). OT-1 TCR transgenic mice [C57BL/6-Tg (Tcra/Tcrb) 1100Mjb/J] were obtained from Jackson Laboratory (Bar Harbor, ME, USA). All the strains were bred in our animal facility under specific pathogen-free conditions. All animal procedures were conducted under institutional guidelines that comply with national laws.

### IFN-γ production assays

CD8 T cells were purified from splenocytes by magnetic sorting using anti-CD8 microbeads (Miltenyi). For polyclonal stimulation, CD8 T cells were cultured (5×10^5^ cells/ml) in complete medium (RPMI-glutamax medium (Invitrogen) supplemented with 10% FCS (Sigma) and 1% penicillin/streptomycin (Invitrogen) together with Concanavalin A (4 ug/mL) (SIGMA), IL-12, IL-2 (10 ng/mL each) (Miltenyi) and irradiated splenocytes (feeder cells) (ratio 1:1). For antigen-specific stimulation, CD8 T cells from OT-1 mice were cultured (5×10^5^ cells/ml) with peptide-pulsed irradiated splenocytes together with IL-12 and IL-2. At day 3, supernatant was harvested and analyzed by ELISA (BD Biosciences) for IFN-γ production.

### *In vivo* experiments

BALB/c mice were subcutaneously injected with 5×10^5^ CT26 melanoma cells. At day 2, 4, 7, 9 upon tumor implantation mice were treated intravenously with 350 pmols/dose (700 peq/dose) of PDL1-blocking antibody (10F.9G2) and 800 pmol/dose of TIM3-Apt1 antagonizing aptamer. Tumor size was calculated by using the following formula: Tumor size (mm^2^) = (length)×(width). Mice with tumor size equaling or exceeding 400 mm^2^ were humanely sacrificed.

## SUPPLEMENTARY FIGURES AND TABLE






