# Supplementary material for: Identification of TIM3 2′-fluoro oligonucleotide aptamer by HT-SELEX for cancer immunotherapy
Source: Oncotarget. 2015 Dec 14;7(4):4522–30. doi: 10.18632/oncotarget.6608 (PMC4826223; doi:10.18632/oncotarget.6608)
Supplement: Supplementary file 1 [file oncotarget-07-4522-s001.pdf]

SUPPLEMENTARY FIGURES AND TABLE

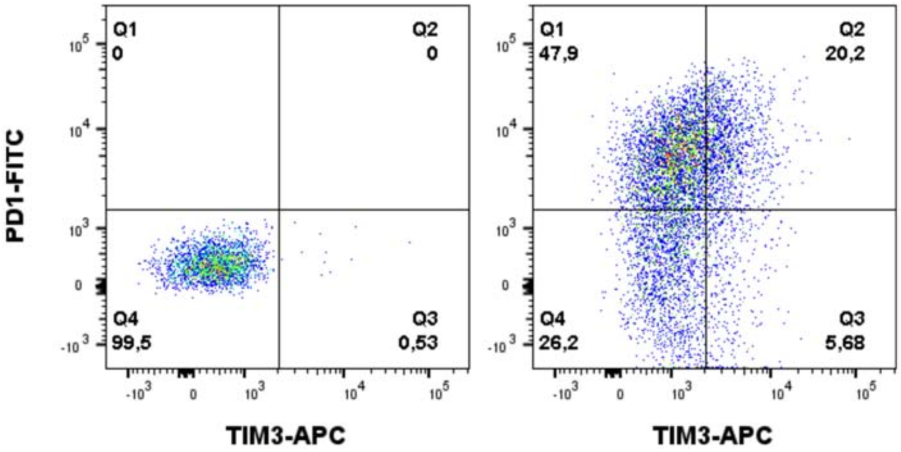

Supplementary Figure S1: Purified CD8 lymphocytes were polyclonally stimulated with ConcA, feeder splenocytes and IL12 and IL2 cytokines. Surface expression of PD1 and TIM-3 on CD8 T cells at day 4 of culture. Dot plots on the left and on the right show cells stained with isotype control mAbs or with the mix of specific antibodies, respectively

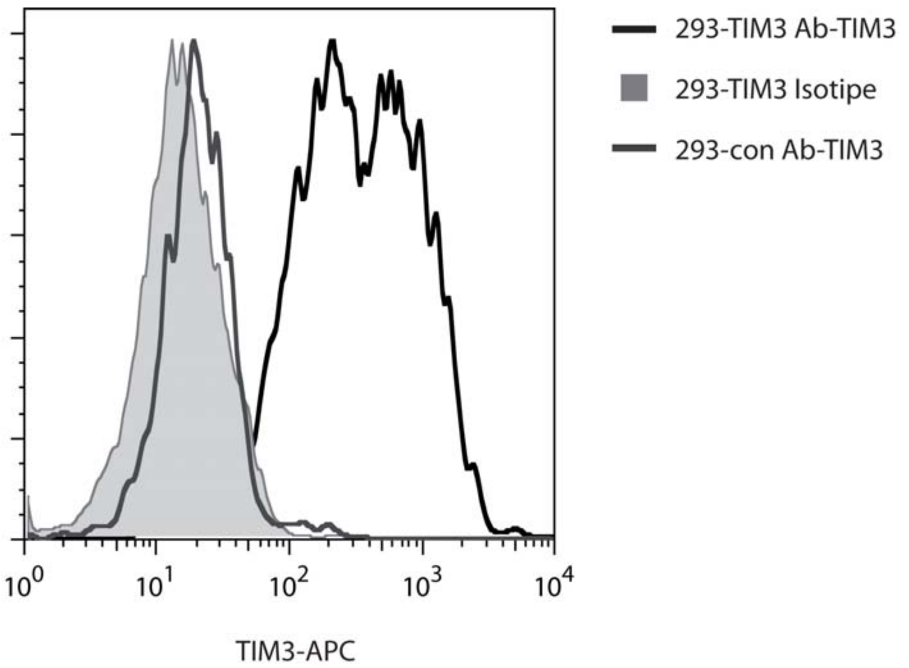

Supplementary Figure S2: Surface expression of TIM3 on 293-TIM3 cells.

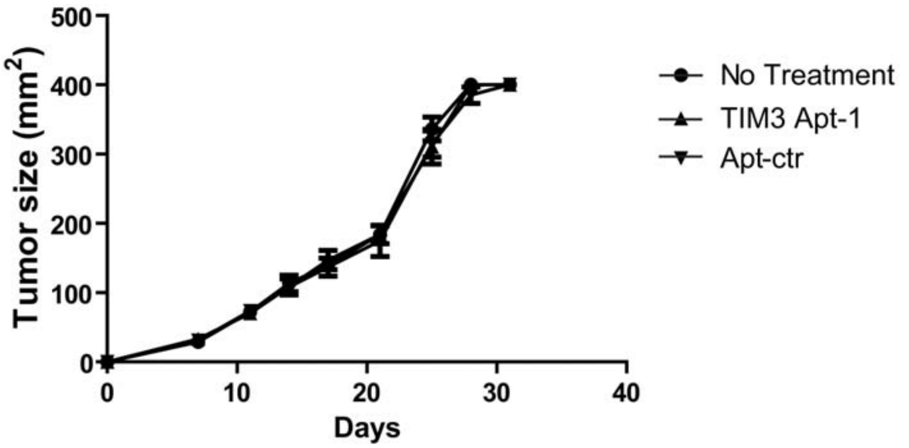

Supplementary Figure S3: Lack of antitumoral effect of TIM3-Apt1 as monotherapy on CT26 tumor bearing mice treated at day 2, 4, 7, 9, 11 and 13 with 800 pmol per injection.

Supplementary Table S1: SELEX conditions

Round	RNA (µM)	TIM3Fc (nM)
1	3	0.8
2	1	0.4
3	0.25	0.4
4	0.125	0.4
5	0.05	0.4
6	0.025	0.2

Supplementary Data 1

See Supplementary Data 1

Supplementary Data 2

See Supplementary Data 2
